# Supplementary material for: Specific patterns of vital sign fluctuations predict infection and enable sepsis diagnosis in pediatric burn patients
Source: PLoS One. 2022 Feb 7;17(2):e0263421. doi: 10.1371/journal.pone.0263421 (PMC8820614; doi:10.1371/journal.pone.0263421)
Supplement: Attachment — Submitted filename: Response to Reviewers.docx [file pone.0263421.s001.docx]

**Response to Reviewers**

**Additional Editor Comments:**

Please address the concerns of the reviewers and we will re-evaluate the paper.

**Reviewers’ Comments:**

**Comments to the Author**

1. Is the manuscript technically sound, and do the data support the conclusions?

The manuscript must describe a technically sound piece of scientific research with data that supports the conclusions. Experiments must have been conducted rigorously, with appropriate controls, replication, and sample sizes. The conclusions must be drawn appropriately based on the data presented.

Reviewer #1: Yes

Reviewer #2: Yes

2. Has the statistical analysis been performed appropriately and rigorously?

Reviewer #1: Yes

Reviewer #2: Yes

3. Have the authors made all data underlying the findings in their manuscript fully available?

The PLOS Data policy requires authors to make all data underlying the findings described in their manuscript fully available without restriction, with rare exception (please refer to the Data Availability Statement in the manuscript PDF file). The data should be provided as part of the manuscript or its supporting information, or deposited to a public repository. For example, in

addition to summary statistics, the data points behind means, medians and variance measures should be available. If there are restrictions on publicly sharing data—e.g. participant privacy or use of data from a third party—those must be specified.

Reviewer #1: Yes

Reviewer #2: Yes

4. Is the manuscript presented in an intelligible fashion and written in standard English?

PLOS ONE does not copyedit accepted manuscripts, so the language in submitted articles must be clear, correct, and

unambiguous. Any typographical or grammatical errors should be corrected at revision, so please note any specific errors here.

Reviewer #1: Yes

Reviewer #2: Yes

5. Review Comments to the Author

**Reviewer #1:** This is an interesting article that highlights the importance and difficulty of diagnosing sepsis in the setting of severe burn injury. I have some concerns for the authors:

Thank you for your insightful comments to add clarity on the study methods and analyses for our article. Please see our comments below.

1. It seems from the data that MAP dips are the most relevant correlation with sepsis. I suggest that a multivariate regression analysis would provide a better insight into the weight or importance of MAP dips relative to other variables.

**Author Response:** We have performed two multivariate regression analyses to determine the weight of each independent variable (fever spikes, HR spikes and MAP dips) relative to the other variables. Our regression analyses found that HR spikes were significant and held more statistical weight in the prediction metric than MAP dips or fever spikes.

Location of Revision: 124-125, 207-211

2. What is the time frame between MAP dips and other variables (fever, HR spikes)? In other words, do these events occur within a similar time frame or do they only occur within the same 24 hour period but do not occur within an hour of each event.

**Author Response:** The variables (fever spikes, HR spikes and MAP dips) all occurred within the same 24 hour study timeframes, however the variables did not occur within an hour of each other.

Location of Revision: 353-355

3. Does the time frame between each variable event correlate to sepsis prediction or accuracy? For example, do events that occur relatively close together (fever, HR spikes, and MAP dips) lead to a more accurate sepsis prediction?

**Author Response:** While we did not detect any immediate trends between the timing of measurements and sepsis accuracy, future studies would benefit from a larger sample size. The exact timing of the measurements depends on a large number of factors and notions in the clinic setting, and therefore it would take a significantly larger patient study sample to be able to analyze such trends. We are currently working on acquiring multi-hospital data sets to achieve such analyses.

Location of Revision: 355-360

**Reviewer #2:** The authors presented a manuscript examining the fluctuation in patterns of simple vital signs data to predict “diagnose” blood stream infection in paediatric burn patients. They included retrospective data from 200 children with >20% TBSA. This work builds on their previously published research of the role of temperature patterns to predict blood stream infection.

I thoroughly enjoyed reading the manuscript, it is well written, referenced, and a good study design despite the package of limitations that comes with retrospective studies. The strength of the study is taking positive blood culture as the “golden standard/reference standard” for defining sepsis rather than other scoring systems that are either not validated or have low reliability. Others have used clinical diagnosis of burn specialist surgeons and critical care physicians as the reference standards for validation to avoid this problem. Furthermore, all these current sepsis scores are difficult to apply to paediatric burns patients. Early diagnosis of sepsis and immediately initiating management was shown to save many lives of our patients. In future, advancement of bedside microbiological sequencing may give all clues that clinicians need for our sick burn patient. However, until then, a simple clinical algorithm would be extremely useful to diagnose sepsis, confidently and reliably. This manuscript is an important step in this path and may lead to a simple global sepsis scoring criteria for early paediatric burn sepsis diagnosis.

I’d congratulate the author on perusing a simple solution for a very difficult problem and I am looking forward seeing the data from their prospective ongoing project. My comments are:

Thank you for your insightful comments to add clarity on the study methods and discussion for our article. Please see our comments below.

1. Title: I fully understand the authors are factual and careful with the title wording. As I personally believe this study would be a step forward in sepsis diagnosis in paediatric burn patients, I would suggest using the keywords (sepsis, diagnosis), to be straight to the point.

**Author Response:** Thank you for bringing this to our attention. We have amended the title of the article to “Specific patterns of vital sign fluctuations predict infection and enable sepsis diagnosis in pediatric burn patients” to further clarify the main objectives of the study.

Location of Revision: 4-5

2. The whole dilemma of burn sepsis scorings is ‘sepsis definition’ and setting ‘Reference Standard/Golden Standard’ to check the reliability of a new scoring system. The authors used Positive Blood Culture (PBC), which is superior to other unvalidated or unreliable scores as definition, however, there are problems regarding interpretations of PBC in clinical practice. Would the author please elaborate why PBC was used and its limitations? Would the authors discuss their views using the “clinical diagnosis of sepsis by burn specialist surgeon and critical care physician” for sepsis definition as reported by Yan et al, 2018, as a reference standard for future trials?

**Author Response:** Positive blood cultures are used at our pediatric burn institution as the current reference standard because they are superior in sepsis accuracy, compared to other unvalidated scoring systems. However, it is also possible to miss infections using blood cultures alone, and the clinical team will often order pan (sputum and urine) cultures for further accuracy.

Before obtaining the positive blood cultures, the clinical team correctly administered empiric antibiotics for 38 patients in the infection group. We were unable to confirm antibiotic use in 11 patients due to limited access to medical records prior to 2011. The clinical team incorrectly administered empiric antibiotics for 35 patients in the control group, which were later confirmed by the routine negative blood cultures. The clinical team correctly diagnosed 11 patients that did not require empiric antibiotics during their inpatient stay.

The Yan et al. study suggests that using clinical diagnoses made by the clinical team as the reference standard is a strong approach for timely identification of sepsis in burn patients. We agree with a similar approach reported by Yan et al., as clinician diagnoses are based on clinical signs of sepsis, such as vital sign fluctuations. The main motivation behind this current study was to develop a rigorous predictive metric that statistically interpreted these clinical signs of sepsis already used by the clinical team to enable sepsis diagnoses.

3. The authors have explained the use of empiric antibiotics after taking blood cultures and the effect on vital signs. Would they please elaborate on how many started the therapy and when, in each group?

**Author Response:** In the infection group, 38 patients were confirmed to be on antibiotics during the study timeframe. We were unable to confirm the use of antibiotics in 11 patients in the infection group due to limited access to medical records prior to 2011. In the control group, 35 patients were confirmed to be on antibiotics during the study timeframe, and 11 patients were confirmed to not require antibiotics during their inpatient stay.

Location of Revision: 301-305

4. It would be interesting to the reader if the authors elaborate on why this simple scoring system has lower specificity than sensitivity? Would that be related to the use of PBC as the ‘Reference Standard’?

**Author Response:** We believe that the simple scoring system has a lower specificity than sensitivity because a few of the patients tend to narrowly miss the cutoff for spikes. It is possible that a longer observation period may increase the accuracy of the cutoff for spikes, which has been the case for predicting infections in our prior work.

Location of Revision: 272-275
